# Perilesional photobiomodulation therapy and physical rehabilitation in post-operative recovery of dogs surgically treated for thoracolumbar disk extrusion

**DOI:** 10.1186/s12917-020-02333-3

**Published:** 2020-04-25

**Authors:** Enrico Bruno, Sara Canal, Michela Antonucci, Marco Bernardini, Federica Balducci, Vincenzo Musella, Matteo Mussoni, Giuseppe Spinella

**Affiliations:** 1 Portoni Rossi Veterinary Hospital, via Roma, 57, 40069 Zola Predosa, BO Italy; 2 Department of Animal Medicine, Production and Health, Clinical Section, University of Padua, Legnaro, Padua, Italy; 3 Department of Health Sciences, University “Magna Graecia” of Catanzaro, Viale Europa - Loc. Germaneto, 88100 Catanzaro, Italy; 4 Centro Veterinario Valmarecchia, via Casale 76, 47826 Villa Verucchio, RN Italy; 5 Department of Veterinary Medical Sciences - University of Bologna, via Tolara di sopra 50, 40064 Ozzano dell’Emilia, BO Italy

**Keywords:** Intervertebral disk extrusion, Thoracolumbar spine, Photobiomodulation, Rehabilitation therapy, Dog

## Abstract

**Background:**

Recent studies have reported contrasting results of the effects of laser therapy on post-operative intervertebral disk herniation, with a lack of evidence-based advantages of this modality within a rehabilitation protocol.

The aim of this study was to report the clinical effects of photobiomodulation therapy within a post-operative rehabilitation protocol in dogs submitted to surgery for thoracolumbar disk extrusion. Twenty-four dogs were included in the study (12 dogs treated with laser therapy and rehabilitation protocol and 12 dogs treated with same rehabilitation protocol but without laser therapy).

**Results:**

All dogs treated with laser therapy showed improved neurological status (Modified Frankel Score more than 3 within 30 days of physiotherapy starting) if deep nociception on admission was maintained (*P* = 0.04). However, Kaplan-Meier analysis did not show any statistical difference in time to regain ambulatory ability, although there was a tendency for a shorter mean time of 14.2 ± 8.55 days in the laser group versus 24 ± 18.49 days in the no laser group.

**Conclusions:**

The use of laser therapy in the post-operative rehabilitation of dogs affected by intervertebral disc extrusion and submitted to surgery for spinal decompression could help improve their neurological status.

## Background

Discopathies, namely protrusion or extrusion of the intervertebral disc (IVD), are neurological conditions affecting the canine spinal cord [1, 2]. Hansen described two types of canine IVD herniation (IVDH): type 1 IVDH, extrusion of the IVD, and type 2 IVDH, protrusion of the IVD [3, 4]. More recently, a non-compressive subtype of IVD extrusion has also been proposed [5, 6]. Compressive IVD extrusion occurs when there is total rupture of the dorsal annulus and massive extrusion of the nucleus pulposus into the spinal canal [7]. It is observed most frequently in chondrodystrophoid breeds and associated with a severe inflammatory response [8, 9]. The dynamic force of compression on the spinal cord from an IVD extrusion and the resultant hemorrhage, vascular compromise, and inflammation are responsible for the ensuing spinal cord damage and the associated neurological signs [10, 11].

Rehabilitation therapy for the neurological patient provides supportive care to protect the patient from complications, and preserve tissue strength and function during the recovery period. Rehabilitation seemed to have a positive influence on the functional recovery of dogs that underwent surgical decompression for any type of IVDH, leading to a shorter recovery time for motor function and limiting the complications related to immobilization and confinement in a cage [12–14].

Photobiomodulation (therapeutic laser), e.g. Low Level Laser Therapy (LLLT), has been investigated in veterinary medicine owing to its potential ability to relieve pain, reduce inflammation, and accelerate the healing process [15, 16]. All these advantages are related to the ability of photobiomodulation to: promote stimulation of cytochrome oxidase in mitochondria with production of adenosine triphosphate (ATP); limit the inflammatory process and leukotriene activity; and improve the release of endorphins and serotonin [17–27]. Several studies have also been conducted to prove the positive effect of LLLT on neural recovery [20, 23, 26]. Photobiomodulation applied to adult rats that underwent T9 dorsal hemisection resulted in significantly increased axonal number and distance of regrowth [26]. However, recent studies in dogs have published contrasting results on the effects of laser therapy on post-operative IVDH; thus the literature lacks clear evidence-based advantages [27, 28].

The aim of this clinical study was to investigate the influence of perilesional therapeutic laser application on ambulatory recovery in dogs submitted to a post-operative rehabilitation program after surgical thoracolumbar spinal cord decompression for IVD extrusion. We hypothesized that the use of perilesional photobiomodulation in the post-operative rehabilitation program would shorten the time to functional recovery.

## Results

Twenty-four dogs were included in the study (Table 1). Group 1 (no perilesional laser) consisted of 12 dogs, 3 males and 9 females. The breeds represented were: Dachshund (*n* = 4; 33.3%), mixed breed (*n* = 3; 25%), Beagle (*n* = 2; 16.6%), French Bouledogue, Poodle, and Cavalier King Charles Spaniel (*n* = 1 of each). The mean age was 5.8 ± 2.57 years (range 4 to 13 years) and the mean body weight was 9.19 ± 3.54 kg (range 3.9 to 16.2 kg). The type of herniation was IVD extrusion (Hansen type I) in all cases. The IVD space predominantly involved lay between the 12th thoracic vertebra (T12) and the 2nd lumbar vertebra (L2), with laterality equally distributed between the right and left. The mean duration of clinical signs before surgery was 6 ± 8.98 days, with 10 dogs having a duration ≤5 days and 2 dogs more than 20 days. The median Modified Frankel Score (MFS - Table 2) on admission to the rehabilitation center was 3 (nine patients had a score of 3, two dogs a score of 2, and one scored 1) (Table 3) and the physiotherapy started in a mean time of 4.3 ± 0.7 days after surgery. Follow-up at 30 days after the start of physiotherapy showed 8/12 dogs (66.6%) with an MFS greater than 3; a similar result was observed after 60 days.

**Table 1:** Clinical cases that met the inclusion criteria

N°	Group	Breed	Sex	Age (years)	BW (Kg)	S-S	MFS T0	T1	T2	MFS T2
1	1	GD	F	4	6.5	1	1	Y	Y	4
2	1	FB	F	4	10.6	1	3	N	Y	4
3	1	Beagle	F	6	12.2	5	3	N	Y	5
4	1	GD	M	13	10.1	2	3	Y	Y	5
5	1	MB	F	6	7	2	3	N	N	3
6	1	MB	F	4	4.9	2	3	Y	Y	5
7	1	MB	M	4	16.2	1	3	N	Y	4
8	1	GD	F	4	4.9	21	3	N	Y	5
9	1	Beagle	F	9	12	30	3	N	N	3
10	1	Poodle	F	5	3,9	3	3	Y	Y	5
11	1	CK	F	5	11.5	3	2	N	N	3
12	1	GD	M	6	10.5	1	2	N	N	3
13	2	MB	M	10	37	15	3	Y	Y	5
14	2	MB	M	7	13	30	3	Y	Y	5
15	2	MB	M	9	30.5	1	3	Y	Y	5
16	2	FB	F	7	12	14	0	N	N	0
17	2	GD	M	6	11	1	3	N	Y	5
18	2	MB	M	11	16	4	3	N	Y	5
19	2	MB	F	15	22	2	3	Y	Y	4
20	2	MB	F	6	16.5	7	0	N	N	0
21	2	GD	M	8	8	1	2	N	Y	4
22	2	MB	M	10	13	10	3	Y	Y	5
23	2	Lagotto	M	8	17	0	1	N	Y	5
24	2	GD	F	7	7.5	2	1	Y	Y	5

Group 1 = no Laser; Group 2 = perilesional laser application; GD = German Dachshund; FB = French Bouledogue; CK = Cavalier King Charles Spaniel; MB = Mixed breed; F = female; M = male; BW = Body Weight; S-S = interval of time, expressed in days, between the appearance of symptoms and surgery; MFS T0 = MFS on admission at rehabilitation center; T1 = neurological evaluation after 14 days of physiotherapy; T2 = neurological evaluation after 30 days of physiotherapy; N = dog not able to ambulate; Y = dog able to ambulate; MFS T2 = MFS after 30 days of physiotherapy. Dogs n.16 and n.20 were discharged with the cart to walk because of their poor prognosis of MFS 0 on admission and no improvement during physiotherapy

**Table 2:** Modified Frankel Score as previously reported by Draper et al., 2012 [27]

Grade 5	Spinal hyperaesthesia only
Grade 4	Ambulatory with paraparesis and /or ataxia
Grade 3	Non-ambulatory paraparesis
Grade 2	Paraplegia with entire superficial nociception in the pelvic limbs
Grade 1	Paraplegia with entire deep nociception in the pelvic limbs
Grade 0	Paraplegia with absent nociception in pelvic limbs

**Table 3:** Distribution of Modified Frankel Score (MFS) at T0 within the two groups. No significant difference was observed

MFS AT T0	Group 1	Group 2	P
3	9/12	7/12	0,92
2	2/12	1/12	
1	1/12	2/12	
0	0/12	2/12	

Group 2 (laser group) consisted of 12 dogs, 8 males and 4 females. The breeds were: mixed breed (*n* = 7; 58.6%), Dachshund (*n* = 3; 25%), one French Bouledogue, and one Lagotto Romagnolo. The mean age was 8.6 ± 2.46 years (range 6 to 15 years) and the mean body weight was 16.95 ± 8.52 kg (range 7.5 to 37 kg). The type of herniation was IVD extrusion (Hansen type I) in all cases. As in group 1 the IVD space predominantly involved lay between T12 and L2, with equally distributed laterality. The mean duration of clinical signs before surgery was 7.25 ± 8.49 days, with 7 patients having a duration of ≤5 days and 5 patients having a duration of 7 to 30 days. The median MFS on admission to the rehabilitation center was 3 (seven dogs presented with an MFS of 3, one dog with an MFS of 2, two dogs with an MFS of 1, and two dogs with an MFS of 0) (Table 3) and physiotherapy started a mean of 4.3 ± 0.8 days after surgery. Excluding the 2 dogs with no deep nociception on admission, all dogs presented an MFS greater than 3 during the 30 days follow-up after starting rehabilitation (Table 1).

In group 1, the mean time taken to ambulate was 24 ± 18.49 days (three dogs started to walk within a period < 10 days), whereas in group 2 (excluding the subjects with an initial MFS equal to 0) the same result was achieved in 14.2 ± 8.55 days (four dogs started to walk within < 10 days).

**Fig. 1:**
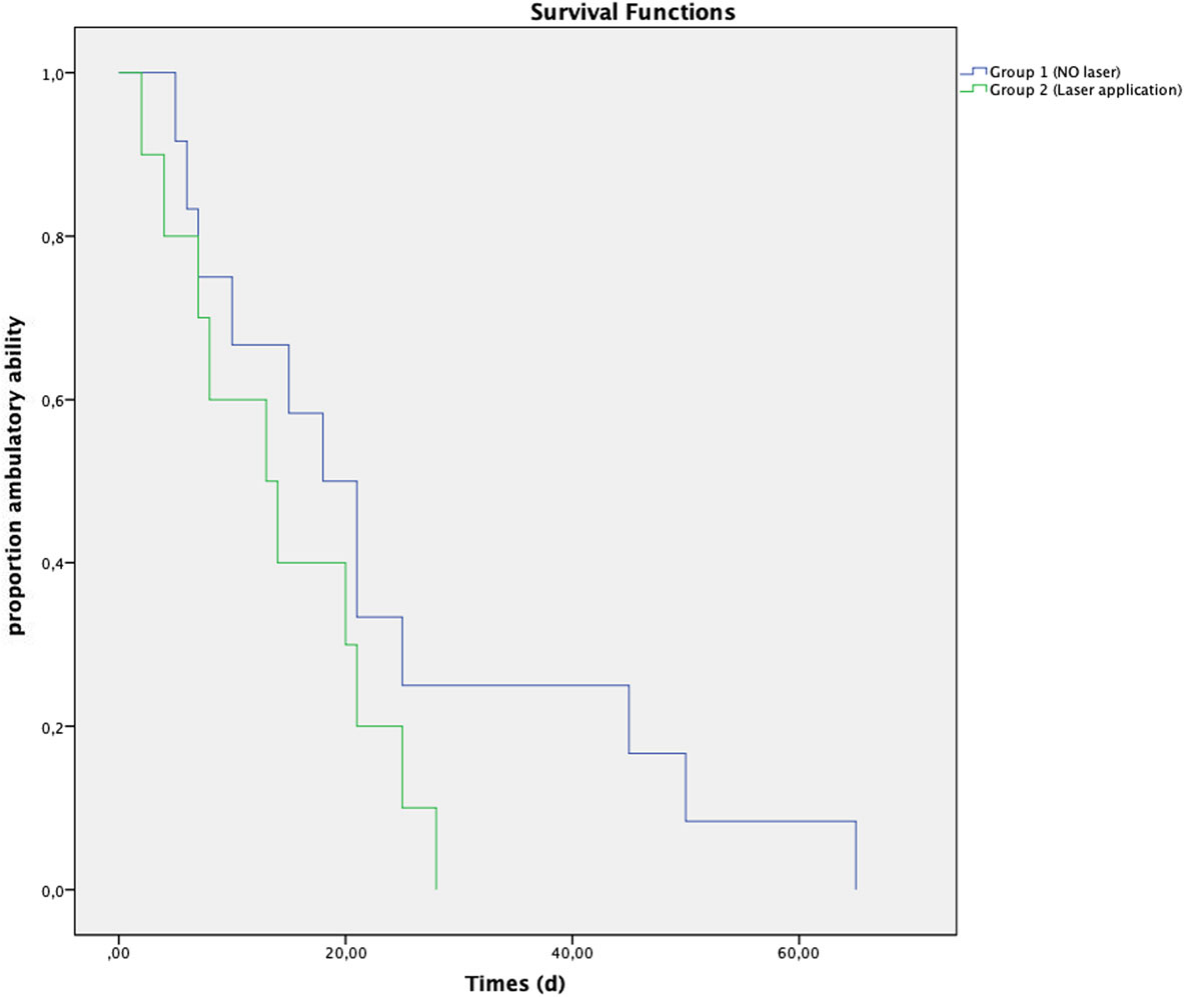
Kaplan-Meier curve demonstrating a non-significant difference between the two groups (excluding the two paraplegic dogs) for time to regain ambulatory ability (P = 0.178)

**Fig. 2:**
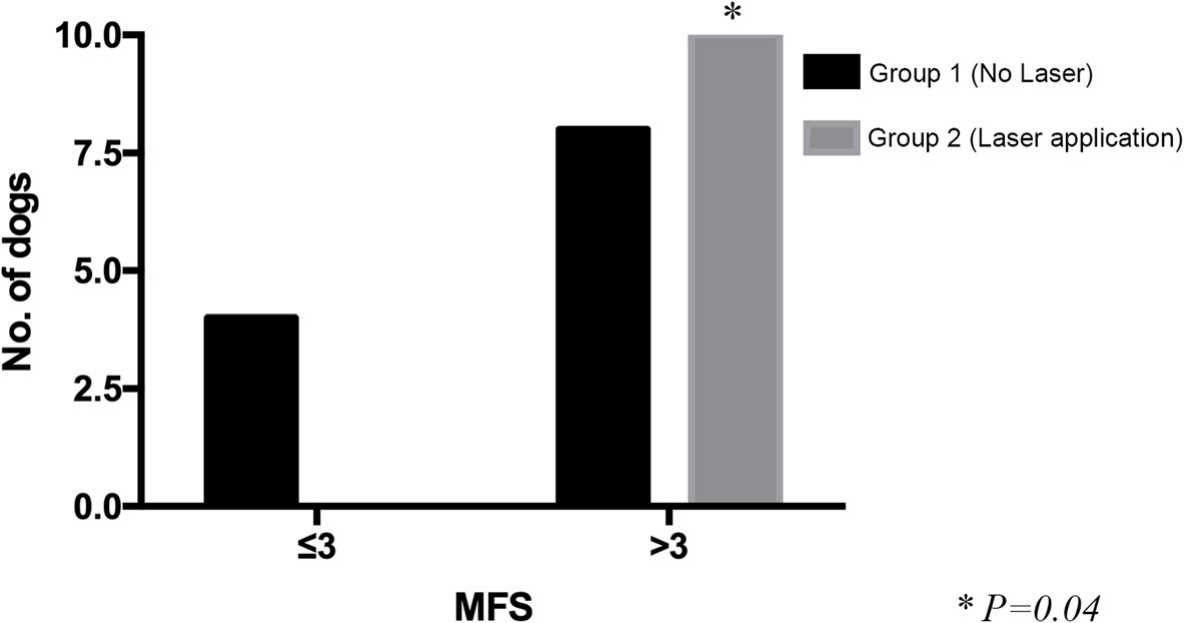
Comparison of MFS at discharge for the two groups. A significant difference was observed with a better result for group 2 (P = 0.04), after exclusion of two paraplegic dogs. (NO. Dogs = Number of dogs)

No statistically significant differences were found for the following variables: sex, MFS on admission (Table 3), time elapsed between the onset of clinical signs and surgery and between surgery and the beginning of physiotherapy, or ability to ambulate on the 14th day of physiotherapy. The statistically significant variables were age (*P* = 0.023) and body weight (*P* = 0.0038). Although there was a shorter mean time to regain ambulatory ability in group 2 (14.2 ± 8.55 days) than in group 1 (24 ± 18.49 days), Kaplan-Meier analysis did not show a statistically significant difference between the two groups (*P* = 0.178) (Fig. 1). After 30 days of the rehabilitation program, a total of 18 dogs out of 24 showed an MFS of at least 4 out of 5 (8/12 dogs in group 1 and 10/10 dogs in group 2, if we excluded those dogs with an MFS of 0 on admission). The difference in MFS between the two groups was statistically significant (*P* = 0.04), with a better result for group 2 (Fig. 2).

## Discussion

The aim of this study was to evaluate the positive effect of perilesional application of therapeutic laser as an integral part of a standard physiotherapeutic protocol in dogs submitted to surgery for thoracolumbar disk extrusion. Our results showed a beneficial effect in dogs treated with laser and with intact deep nociception on admission (all these dogs gained a final MFS ≥4); however, they failed to demonstrate a significant difference in speed of recovery, despite a shorter mean time of 14.2 ± 8.55 days for ambulatory recovery in the laser group, compared to a mean time of 24 ± 18.49 days in the no-laser group.

Of the 24 dogs included in this study and divided into two therapeutic groups (12 dogs in each group), 8 and 5 belonged to chondrodystrophic breeds in group 1 (66.7%) and 2 (41.7%), respectively. The inclusion of various breeds was in line with other publications, although a higher prevalence of chondrodystrophic breeds was previously reported (86% in Draper et al. [27], 70% in Bennaim et al. [28]). However, based on the retrospective nature of our investigation, we were unable to correctly classify (as chondrodystrophic or non-chondrodystrophic) 10 mixed breed dogs (three in group 1 and seven in group 2), which represented 42% of our study population, or to conduct a robust evaluation of chondro- versus non-chondrodystrophic distribution.

The variable “age” of the dogs was also not homogeneously distributed within our study. In group 1, the mean age was 5.8 years, whereas in group 2 it was 8.6 years. Although IVD degeneration-related diseases can occur at any age, the incidence rate increases with age as determined in a sample of various breeds of dogs aged < 12 years [1]; and a peak incidence around the age of 4–6 years was previously reported in chondrodystrophic breeds [29]. The variable age could be a limiting factor in the functional recovery of older dogs [25, 30]. However, this hypothesis was not corroborated by a recent publication [31], which investigated only one type of breed (dachshund) with a mean age of 5.9 years. Moreover, group 2 included heavier dogs (mean weight 16.95 kg) than group 1 (mean weight 9.19 kg). High body weight could also interfere with and have a negative effect on functional recovery, since heavier patients are more complicated to manage, especially in the manual therapy session and in passive exercises, and have more difficulty in regaining ambulatory capacity without any support [2, 27, 32]. However, our results showed that heavier patients, retaining intact deep pain perception (more prevalent in group 2), achieved an MFS ≥ 4 at discharge. This result could be related to the beneficial effects of laser therapy applied in group 2, and it could explain why, although all dogs had a minimum MFS of 4 at discharge, the time taken to regain ambulation did not differ significantly from group 1, which on average included younger and less heavy dogs.

The rehabilitation program was homogenous for the types of exercises (passive and active) and their succession within the two groups; differences were only related to progressive increases in frequency (number of repetition) and resistance (i.e. variation in height of water for underwater treadmill) on the basis of the clinical progression of each patient during the period of rehabilitation. Follow-ups were carried out at 14, 30, and 60 days from the beginning of rehabilitation with the aim of evaluating the short- and long-term therapeutic effects of photobiomodulation. In the short-term, application of laser therapy has a predominantly anti-inflammatory and vasoactive effect [33]. In the medium- and long-term, it can act positively on relieving neuropathic pain [33]. A phase-controlled and progressive rehabilitative protocol was previously proposed by Bennaim et al. [28], and included only one conclusive clinical follow-up at 10 days from the beginning of the treatment with the aim of evaluating only short-term laser therapeutic effects. No differences in recovery-related variables among groups were observed. In contrast, Draper et al. [27] limited their study to laser application (once a day for 5 days) and basic physical rehabilitation exercises (passive range of motion, supported standing and toe-pinch withdrawal). They monitored patients until they were able to ambulate autonomously, observing a median recovery time of 3.5 days in their laser group, and of 14 days in the control group. As reported by Draper et al. [27], time to ambulation has previously been considered as a measure to demonstrate the efficacy of laser therapy. Furthermore, they monitored patients until they were able to ambulate autonomously (minimum MFS achievement of 4) [27]. As reported by Bennaim et al. [28], our patients showed a mean recovery time greater than 10 days. This parameter was greater than the 3.5 or 14 days reported by Draper et al. [27]; but in comparison to those authors, a similar difference in recovery time (10 days) was observed between group 1 (mean time of 24 days) and group 2 (mean time of 14 days). In our opinion the faster recovery time previously reported could be associated with the earlier application of LLLT (immediately after surgery) [27, 28].

Although the difference in time taken to regain ambulatory ability in our two groups was not statistically significant, a shorter mean time was recorded in group 2 (14.2 ± 8.55 days) than in group 1 (24 ± 18.49 days). Moreover, in the current study, the only two dogs in group 2 that did not improve were paraplegic (MFS 0). As previously reported, patients lacking deep nociception generally have a worse prognosis for regaining nociception and, consequently, the ability to walk, with a probability of success of about 60% [34, 35]. For this reason, the two paraplegic dogs without nociception included in group 2 were excluded from the Kaplan-Meier test in order to avoid bias in overall survival observation. Conversely, patients with thoracolumbar spinal cord compression and intact deep pain perception that received adequate and timely surgical and physiotherapeutic treatment had a good/excellent prognosis (probability of success > 90%) [35, 36], with a mean time to recover voluntary ambulation within 10 or 13 days [37, 38]. More recently, Hodgson et al. [39] performed a retrospective study on 248 dogs with an MFS ≤3 with the aim of comparing dogs receiving in-house rehabilitation with a control group. They unexpectedly observed that, despite a lower complication rate in the rehabilitation group, normal conscious proprioception and ambulation returned earlier in the control group (42 days and 14 days, respectively) compared to the group submitted to rehabilitation (49 days and 28 days, respectively) [39]. Similarly, patients included in our study with intact nociception presented a mean recovery time to ambulation greater than 10 days, highlighting that dogs admitted to a rehabilitation center may experience inherently slower recovery. However, in our study group 1 showed a mean time to ambulation of 24 ± 18.49 days (only three dogs started to walk within a period < 10 days), whereas for group 2 the same result was achieved in 14.2 ± 8.55 days (only four dogs started to walk within a period < 10 days).

Although these results indicate a partial beneficial effect of laser therapy on neurological recovery, this retrospective study was limited by several technical aspects. One limitation was the number of subjects included in the statistical analysis, because a larger sample would better discriminate the differences in gait recovery between the two groups. Other limitations were related to: the lack of a control group that did not receive any rehabilitation treatment following surgery; the absence of homogeneity in animal body condition; differences in owner compliance; and a potential difference in the skill set of the rehabilitation staff (although specialized), because of the lack of randomization in laser application within the study population.

## Conclusion

Our results support the benefits of the use of perilesional laser therapy within a rehabilitation protocol for neurological recovery in dogs affected by intervertebral disc extrusion and submitted to surgery for spinal decompression. However, further prospective investigations with greater standardization of enrolled clinical cases and a long-follow-up evaluation are greatly needed.

## Methods

All records of dogs with neurological signs consistent with a diagnosis of thoracolumbar IVD extrusion and referred to the Unit of Physiotherapy “Kinetic” Center of “I Portoni Rossi” Veterinary Hospital from April 2017 to December 2018 were evaluated. The criteria for inclusion in this retrospective study were as follow:

- Neurological exam performed by a board-certified neurologist of the European College of Veterinary Neurology (ECVN) or a senior resident of the ECVN;
- Diagnosis of thoracolumbar IVD extrusion obtained by magnetic resonance imaging study of the spine, using standard and previously described imaging criteria [40];
- Decompressive surgery, hemilaminectomy with or without fenestration of the affected IVD, performed by a board-certified surgeon (ECVN Diplomate);
- Post-operative in-house physiotherapy for a minimum period of 14 days;
- MFS on admission to the rehabilitation center equal to or less than 3 (Table 2) [26].

Dogs suffering from any concurrent disease that could interfere with locomotion or post-operative recovery were excluded from the study.

The following data were also recorded for each patient: breed; age (years); sex (male or female); weight (kg); site of IVD extrusion; duration of clinical signs before surgery; MFS on admission to rehab center; regained ambulation ability or not after 14, 30 and 60 days from start of rehabilitation; and the total number of days to regain ambulation. As previously reported, patients were considered ambulatory when they achieved an MFS of 4, that is they could stand and take three steps on a nonslip surface without falling in the absence of physical manipulation [27].

After decompressive surgery, all dogs included in this study received the same protocol of analgesia: methadone (0.2 mg/kg IM - Semfortan, Dechra, Eurovet Animal Health BV, AE Bladel) was administered every 4–5 h for the first 24 h after surgery, and meloxicam (0.1 mg/kg orally - Metacam, Boehring Ingelheim Vetmedica GmbH – Germany) was also administered once a day for 4–5 days. After 24 h, tramadol (2–3 mg/kg IM or orally, three times daily - Altadol, Formevet s.r.l., − Milano, Italy) was administered if needed (usually for 2–3 days), e.g. when the dog showed clear signs of pain during daily management (walking outside to urinate or defecate) or during physiotherapy manipulations.

The post-operative rehabilitation protocol was similar for all dogs included in the study, starting within 5 days after surgery and continuing for at least 14 days, unless the dogs achieved an MFS of 4 before this time period, as previously planned with the owner. Dogs were managed for rehabilitation twice a day (5 days a week), brought outside 3–4 times daily to urinate and defecate, and confined in a cage for the remaining time.

In more detail, the rehabilitation protocol, with increasing intensity, included: soft tissue treatment to decrease muscle spasms and relax the dog [41], passive range of motion (PROM) (flexion and extension of each joint of each pelvic limb, 15 repetitions), limb stretching, and in the advanced rehabilitation phase assisted proprioception and balance exercises, initially using a support, sit-to-stand exercises and cavaletti rails. From the 7th day after surgery (after a surgeon’s evaluation of surgical scarring), patients exercised daily using an underwater treadmill, starting with a low velocity, 2–3 min per day, with the water level set at the height of the greater trochanter of the femur. The underwater treadmill velocity and session duration were gradually increased, depending on the recovery status and resistance of the dog.

Dogs were retrospectively divided into two groups according to whether they received (group 2) or not (group 1) a perilesional application of photobiomodulation. The distribution of dogs into one of the two groups coincided with the acquisition of the Class IV therapeutic laser diode device (MPHI Vet Orange, ASA Laser, Arcugnano – Italy), power up to 1.2 W, Peak Power 75 W and Target Area Ø 2 cm highlighted by high-efficiency red light LEDs. The laser treatment was applied with a pre-set program for IVD disease in dogs, using the Multiwave Locked System (MLS®) (808 nm and 905 nm-wavelength continuous and pulsated, synchronized and combined emissions), with 50% of duty cycle, 18 Hz of frequency and an energy density of 4 J/cm^2^. Based on the manufacturer’s recommendations and previous publications [27, 28], laser therapy was applied transcutaneously, in contact with shaved skin with a latero-median probe inclination of 45° and at six different points (three on both sides): one point coincided with the spinal segment associated with hemilaminectomy, and the two other points on adjacent spaces (cranially and caudally to the surgical site). The duration of laser therapy was scheduled and preset by the machine at 2′30″ (cumulative dose: 75.292 J) or 3′20″ (cumulative dose: 75.389 J) minutes, in relation to the patient’s skin color (light or dark skin/coat, respectively). These two different durations were previously set by the manufacturer in order to ensure the same therapeutic effect on different dogs. Photobiomodulation therapy was performed once a day, before therapeutic exercises and by specialized veterinary operators (Certified Canine Rehabilitation Practitioner – CCRP licensed). Moreover, the neurologists, who were blinded to the therapeutic protocol, periodically re-evaluated (every 2–3 days) all the patients to avoid potential observer bias.

The rehabilitation program was considered completed if performed for a minimum of 14 days in-house at the clinic and by a CCRP physiotherapist. However, if requested by the owners and vets, it was continued until 30 or 60 days after surgery.

Because of the retrospective nature of this clinical study, no experimental protocol was performed on any animal. All procedures were approved by the owners and were performed during routine clinical examination carried out by certified licensed veterinarians following a normal standard protocol and guidelines accepted in the veterinary clinical practice by the National Federation of Italian Veterinarians (FNOVI Deontological Guidelines, art.15).

### Statistical methods

The recorded data were submitted to descriptive statistical analyses, reporting the mean, range, and standard deviation of the recorded data. Clinical variables were then compared using a Chi-Square test, Fisher exact test, and T test. A Kaplan-Meier test was performed to evaluate the recovery time to autonomous ambulation. Statistical analysis was accomplished with STATA 10.0 (Stata Corp., College Station, TX, USA), and significance was set at *P* < 0.05.

## Data Availability

All data generated or analyzed during this study are included in this published article. Any other information is available from the corresponding on reasonable request.

## Abbreviations

IM: Intramuscular
IVD: Intervertebral disc
IVDH: Intervertebral disc herniation
LLLT: Low-level laser therapy
MFS: Modified Frankel Score
